# Indian egg donors’ characteristics, motivations and feelings towards the recipient and resultant child

**DOI:** 10.1016/j.rbms.2016.04.003

**Published:** 2016-05-11

**Authors:** V. Jadva, N. Lamba, K. Kadam, S. Golombok

**Affiliations:** a Centre for Family Research, University of Cambridge, Free School Lane, Cambridge, CB2 3RF, UK; b Corion Fertility Clinic, Trans Avenue, Lokhandwala Road, Andheri (West), Mumbai–400053, India

**Keywords:** characteristics, Indian egg donors, motivations, recipients

## Abstract

This is the first study to examine characteristics, motivations and experiences of Indian egg donors. In-depth interviews were conducted with 25 egg donors who had donated during the previous 8 months at a fertility clinic in Mumbai. The semi-structured interviews were conducted in Hindi and English. In addition to demographic information, data were collected on donors’ motivations for donating, with whom they had discussed donation, and feelings towards the recipients. The response rate was 66%. All participants were literate and had attended school. Twenty (80%) egg donors had children and five (20%) did not. The most common motivation (19, 76%) for donating was financial need. Egg donors had discussed their donation with their husband or with close family/friends, with almost all mentioning that wider society would disapprove. The majority (20, 80%) had no information about the recipients and 11 (44%) preferred not to. The findings highlight the similarities and differences between egg donors from India and those from other countries and that egg donors are of a more varied demographic background than surrogates in India. Given that India has been a popular destination for fertility treatment, the findings have important implications for regulation and practice within India and internationally.

## Introduction

It is estimated that approximately 70 million couples worldwide experience infertility and that, of these, roughly 40 million seek fertility treatment (Boivin et al., 2007). Egg donation is used by infertile heterosexual couples when the woman is unable to produce good-quality eggs herself. It is also increasingly used by male couples and single men to achieve fatherhood: the egg donor provides the egg, which is fertilized by the father’s sperm using IVF and the resulting embryo is gestated by a surrogate. India was a popular destination for gay male couples seeking surrogacy up until the change in regulation in 2012, which no longer permits male couples and single men to access Indian surrogacy (Jadva, 2016). There are no official statistics available on the number of egg donation cycles carried out in India. However, according to the National Assisted Reproductive Technology (ART) Registry of India, between 2007 and 2009 the number of anonymous egg donation cycles doubled, from 1047 to 2130 (Malhotra et al., 2013). This increase could be related to Indian patients becoming less concerned about using donated gametes (Widge and Cleland, 2011) and to the growing number of fertility clinics treating international patients (Gupta, 2012). The UK has seen a rise in the number of couples who travel abroad for fertility treatment (Crawshaw et al., 2012), although there are no official statistics available on its prevalence. A survey of Canadian and US clinics found that patients travelling abroad from the US for fertility treatment were most likely to travel to India and Asia, with 41% pursuing standard IVF and 52% looking for IVF with donor eggs (Hughes and DeJean, 2009). Reasons for travelling abroad include particular treatments being unavailable in the country of residence, either because of legislation, a lack of expertise, or because potential patients do not meet the criteria for receiving treatment (e.g. because of age) (Ferraretti et al., 2010). In addition, donor gametes may be unavailable in the country of residence, and success rates, waiting times and cost may be better elsewhere (Blyth, 2010).

The Indian Council of Medical Research (ICMR) does not allow known donation, i.e. donation by a friend or relative of the couple, although some clinics do offer this service to patients (Malhotra et al., 2013, Widge and Cleland, 2011). Egg donors must be aged between 21 and 35 years and may receive financial compensation for their donation, which may be a significant sum when compared with the amount that can be earned through other forms of work (Gupta, 2012). Financial remuneration has been found to be an important motivation for egg donors who donate in countries where payment is permitted, such as the USA, although this is often cited alongside altruistic motives (Almeling, 2011; Kenney and McGowan, 2010; Lindheim et al., 2001, Purewal and van den Akker, 2009). It has been argued that it should not be assumed that having financial motives for donating eggs prevents egg donors from being motivated to help others, as egg donors are likely to have more than one reason for donating (Pennings et al., 2014). Nevertheless, studies have found that egg donors who receive greater sums of money are also more likely to state financial motives (Lindheim et al., 2001, Pennings et al., 2014).

Egg donors in the USA, Canada and UK have been found to detach themselves emotionally from their eggs by viewing their donation as ‘just an egg’, which helps recipients to have ‘their own child’ (Almeling, 2011, Blyth et al., 2011, Graham et al., 2016). Studies have also found that some egg donors want information about the outcome of their donation, specifically whether or not their donation had led to the birth of a child (Graham et al., in press; Kenney and McGowen, 2010; Purewal and van den Akker, 2009), although few donors receive information about this in practice.

In India, couples using gamete donation largely keep this a secret, not only to conceal their infertility and shield themselves from the negative social stigma associated with it, but also to protect the perceived biological connection between the married couple and their child (Bharadwaj, 2003, Widge and Cleland, 2011). A study of the perceptions of gamete donation amongst Indian ethnic minority people living in the UK similarly found that gamete donation was often hidden from others owing to a fear of negative repercussions for the family and child (Hudson and Culley, 2014). One means of concealing the use of donor gametes was by finding a donor who was closely matched to the couple in terms of physical characteristics. However, in the UK there is a lack of donors from ethnic minority groups (Human Fertilisation and Embryology Authority, 2014), and Asian women have been reported to be less willing than Caucasian women to donate their eggs (Purewal and van den Akker, 2006).

Although there has been a great deal of interest in the experiences of surrogates in India, much less attention has been paid to the women whose eggs are used for surrogacy pregnancies. When Indian egg donors donate to international patients who are not of Indian ethnicity, the child may look different to his/her parents; this may make the parents more likely to disclose their child’s donor conception to them. These children will be unable to obtain the identity of their donor should they wish to, as Indian egg donors donate anonymously. For children born to UK patients, this will be in direct contrast to their counterparts conceived at UK clinics, who will be able to access the identity of their donor when reaching adulthood. As very little is known about women who donate their eggs in India, this study aimed to investigate the characteristics, motivations and experiences of Indian egg donors, including who they discussed their donation with and their feelings towards the recipients and resultant child. This investigation will not only provide information for professionals and policy makers but will also inform future directions for study.

## Materials and methods

Egg donors recruited to the study were from the Corion Fertility Clinic, Mumbai, and were originally referred to the clinic by an egg donor agency. The clinic is a leading fertility clinic in Mumbai and performs approximately 100–120 egg donation cycles per year, of which 60–70% are part of surrogacy arrangements. Approximately 60% of patients are from overseas, mainly from Australia, the USA, the UK, Israel and Ireland.

All egg donors who had donated at the clinic in the 8 months prior to interview were eligible for this study. In total, 46 egg donors were identified, of which eight could not be contacted. Of the 38 contacted, 25 agreed to take part, representing 66% of those contacted and 54% of all eligible egg donors. Semi-structured interviews were conducted in a private room at the clinic. The interviews were conducted in Hindi and English by NL who later translated the Hindi into English and transcribed the interviews for analysis. Data were obtained on egg donors’ demographic characteristics, their reasons for donating, including where they had first heard about egg donation, their understanding of egg donation, with whom they had discussed egg donation and how they felt towards the recipient and future child. Egg donors received Rs 1000 (£10) for taking part in this study. Ethical approval for this study was obtained from the University of Cambridge Psychology Research Ethics Committee and from the Corion Fertility Clinic Ethics Committee.

Transcripts were systematically analysed by creating a list of codes to classify participant’s responses to the different variables under study. All transcripts were rated according to these codes. Data are presented as number of cases and percentages. In addition, excerpts from the interview data are presented to help illustrate the quantitative data.

## Results

### Demographic characteristics

Twenty-five egg donors took part in this study. Egg donors were aged between 21 and 31 years (mean 25 years). Eleven (44%) were Hindu, 11 (44%) were Muslim, two (8%) were Sikh and one (4%) was Christian. Thirteen (52%) egg donors were married, nine (36%) were either divorced or widowed and three (12%) had never been married. Twenty (80%) had children of their own and five (20%) did not. Their children ranged in age from 2 to 12 years. Twenty-two (88%) egg donors had donated more than once and 3 had donated only once. The maximum number of previous donations (across different clinics) was 7.

All 25 participants were literate and had attended school. Five (20%) had completed a BA degree. Eighteen (72%) women were currently working. Their occupations varied greatly and included manual and non-manual jobs, with income ranging from Rs 1500 (£15) to Rs 40,000 (£400) a month (median = Rs 8000 (£80). Monthly household income ranged from Rs 9000 (£90) to Rs 100,000 (£1000). Some reported that income was not regular and therefore varied from one month to the next. The majority (17, 68%) were bilingual or multilingual and all spoke Hindi. Other languages spoken included Marathi, Gujurati, English, Tamil, Nepali, Punjabi and Bengali.

### Motivations

The most common primary motivation given by 18 (72%) egg donors was financial need, with only one egg donor saying that she wished to help a childless couple. Six (24%) mentioned financial motivations together with wanting to help others, for example:

‘And then, of course the money is tempting. But at the end of the day, when you think about it, it’s also kind of helping something, you know. Somebody can have a baby and probably have a better life because of that. And I know a lot of women feel that their life has come to an end if they can’t conceive and they are not women enough if they can’t conceive and that is extremely sad. So when you think about it, you know, when you put the whole money thing aside and you only think about it from another lady’s point of view, so I think you feel nice that you are in that position… So I basically feel I am very much of a feminist. So if it comes to doing something for another woman, I’ll be more than willing to help that lady.’ [Spoken in English.]

‘We get payment. But some people see it only from the money perspective. And some people view it from a humanity perspective. The fact that it helps someone beyond me getting money makes me happy.’ [Translated from Hindi.]

In terms of what the payment was spent on, some egg donors gave more than one response. Ten (40%) egg donors reported that the money was spent on their own child(ren): for example, for school fees. Seven (28%) had used the money to pay rent and five (20%) had used the money to repay debts. Ten (40%) egg donors had spent the money on other items, including paying a lawyer for divorce proceedings, putting the money into savings and buying gifts for themselves or their family.

### Hearing about egg donation

The majority (21, 84%) of egg donors had first heard about egg donation by word of mouth from family members, friends, neighbours or agents. Agents were usually previous egg donors who received payment for finding new egg donors. The person from whom participants first heard of egg donation had almost always been an egg donor themselves.

Those egg donors who were from a higher social class (6, 24%) were more likely to have either already known of egg donation (2, 8%) or responded to an advertisement in a newspaper (2, 24%). One said that a recent Bollywood movie *Vickie Donor* (a movie about a sperm donor set in Mumbai) had made her think about donating her eggs. These more-educated women also researched the procedure on the internet, which provided information without compromising anonymity, as this egg donor described:

‘I read about it online before I got into it. I didn’t really take anybody’s opinion or advice because I didn’t want anybody to know about it because I really don’t think people are going to, you know...it’s not going to go down very well with people. I think our society is very conservative and even though a lot of people do it, they don’t want to talk about it. So I’ve not really mentioned it to anyone so I just read a little bit about it online. It seemed ok.’ [Spoken in English.]

### Payment

Most (19, 76%) egg donors received a total of Rs 25,000 (£250) for their donation. Six (24%) had received more than this amount (four of whom had a higher educational qualification), with Rs 60,000 (£600) being the highest amount reported. Six (24%) egg donors stated that they would not be egg donors if they did not receive money for it, 10 (40%) were unsure and seven (28%) said that they would donate eggs without receiving payment, particularly if the recipient was poor or had financial difficulties. Two (8%) did not say.

### Initial feelings about egg donation

Three (12%) women reported initially feeling disbelief about the possibility of egg donation: ‘I was not convinced at first that something like this could happen.’ [Translated from Hindi.]

Six (24%) egg donors reported that initially they had felt scared, for example:

‘I was wondering how it would happen. I also took a lot of information and then I went ahead with it. I was only scared that later it should not affect me from getting pregnant’. [Translated from Hindi.]

For two (8%) of the egg donors, being paid for something that involved little work had led them to initially feel that egg donation must be wrong:

‘It felt wrong at some level. As if we are selling something for money. If someone’s life is getting better because of me, that is a different thing. But taking money for it felt wrong to me. First time I felt this. However, when I did it again, I thought I am also benefitting from it and so are the clients. That is why I did it.’ [Translated from Hindi.]

### Understanding of egg donation

The extent to which egg donors could express their understanding of egg donation varied greatly. Two (8%) egg donors (both highly educated) spoke coherently and used scientific language to explain their understanding of egg donation. Three (12%) egg donors were aware that eggs were taken from them but did not say what the eggs were used for. These egg donors reported that either their agents had not told them or that they had not asked. Indeed, one questioned why she would need to know what the eggs were used for. Most (19, 76%) egg donors were aware that eggs were retrieved from them and that these were used to help create babies for infertile couples:

‘They take it out of our body and from what I have heard, when someone doesn’t have kids because of some issues, then they use our eggs, transplant in them to facilitate pregnancy. I think that’s the case though I am not very sure.’ [Translated from Hindi.]

One egg donor had been a surrogate. Four (16%) were planning to be a surrogate in the future, 17 (68%) said they would not be a surrogate and three (12%) were unsure.

### Telling others

The vast majority of participants had not told many people about their role as an egg donor, with two having not told anyone. All married egg donors had discussed the donation with their husband (by Indian law, egg donors must obtain their husband’s permission to donate), and often the husband accompanied the egg donor on visits to the clinic. Seven (28%) mentioned that they had told friends. Whilst 13 (52%) participants had told other family members, usually their sisters or mother, others had decided not to tell wider family:

‘No. I don’t want to show it and tell anyone about it. Because I have children and I fear someone would tell them about it in a bad way. Then people will say that your mother did such things. I don’t want to fall in the eyes of my children for anything.’ [Translated from Hindi.]

People’s lack of understanding about egg donation was often reported as the main reason for keeping it a secret:

‘Actually if you see, egg donation is a good job. But society will not understand, they will say you are selling your child, you are doing something wrong, because society is not yet so developed. Egg donation is good as well as bad, when we are donating eggs, someone will benefit by having babies. But society has a different mentality. They will taunt us and find fault with us. In my neighbourhood, they think of it as selling our baby.’ [Translated from Hindi.]

Whilst egg donors spoke of negative societal attitudes, 13 (52%) viewed egg donation in a positive light, stating that it benefitted others and also helped them by providing money:

‘I feel that I am doing something good for someone else. It’s helplessness for us and a necessity for them. These two come together.’ [Translated from Hindi.]

‘Yes. and I also want people to be a little more open-minded about this. This is not a bad thing. In fact, in our society there are a lot of bad things that are happening and no one says it is bad. So if this is helping someone I don’t think it should be considered as bad.’ [Spoken in English.]

### Feelings towards recipients

Most egg donors (20, 80%) had no information about whom their donation would help but were generally aware that their eggs would help couples to have a baby. Egg donors expressed good wishes towards the recipients and hoped that their treatment would be successful:

‘I will feel very good that they get it. I actually will go pray for them in the temple. I will pray, that god give them (child). What he/she wants, he/she gets. If they have spent so much money on me then whatever it is. So I wish god gives them. That is what I will think.’ [Translated from Hindi.]

Thirteen (52%) women said they would wish to meet the patients if asked and 11 (44%) did not want any information about the patients. For example, one said ‘I feel like not knowing is better’. [Translated from Hindi.] One did not answer.

### Feelings towards resultant child

Fifteen (60%) of the egg donors would be happy to meet the child if asked and six (24%) did not want information about the child or had no interest in meeting the child, for example: ‘No, no. Not really. Somebody has to send me a picture, I’ll say ok. Nice looking baby.’ [Spoken in English] Three (12%) said that they would like to know if a child had been born and 1(4%) did not say.

Women were asked if they felt there was a possibility that the child could look like them. Twelve (48%) said they were unaware of this, six (24%) said that it was possible and one said it might be possible. Six (24%) were not asked.

In terms of telling the child about the egg donation, 10 (40%) felt that the child should not be told, four (16%) said that they should be told and one (4%) said it was up to the parents. The remainder (7, 28%) had no opinion about this.

## Discussion

This is the first study of women in India who donate their eggs. The findings highlight the similarities and differences between egg donors from India and those from other countries. The majority of egg donors reported payment as their primary motivation; however, others also valued being able to help someone in need, with some mentioning that they would be willing to donate eggs even without receiving payment. That financial gain was the main motivation is not surprising, and has been found amongst egg donors from other countries where compensation is permitted (Almeling, 2011, Pennings et al., 2014). For those women in this study who also had reported that they wanted to help someone, this was perhaps related to their understanding of the social stigma associated with childlessness in India, as illustrated by the egg donor who felt she was helping to remove the blame and burden of infertility for another woman.

All egg donors were aware of the stigma associated with egg donation. Yet, feeling that they were doing a good deed led some of them to believe that egg donation was not ‘wrong’. Being an egg donor was a secret shared only with trusted confidants, just as recipients of eggs in India and their immediate family keep the use of donor eggs confidential (Bharadwaj, 2003). However, even in the UK, where open-identity donation is practised (i.e. the resultant child can have access to the donor’s identity on reaching adulthood), egg donors have been found to be selective about whom they discuss their donation with and were found to give similar reasons; for example, that others will not understand and may view it as giving away their children (Graham et al., in press). In the present study, only those egg donors who were agents were more open about their role, as this openness helped them advertise egg donation and recruit new donors.

The money earned from egg donation was usually spent on children and paying household bills. Studies of Indian surrogates have similarly found that money received from surrogacy is used for children’s schooling (NL, unpublished data). Egg donors in this study were of higher socioeconomic status compared with surrogates at the same clinic, with median monthly incomes of Rs 8000 (£80) and Rs 3500 (£35), respectively (NL, unpublished data). Most egg donors were unwilling to be surrogates, suggesting that these two groups of women (egg donors and surrogates) are distinct, with the demarcation possibly based on socioeconomic status. The study also found that the amount of money received varied between egg donors, with some of those with a higher educational qualification being paid more. This is a similar finding to those from other countries where payment is permitted, e.g. the USA, where egg donors who are more desirable (because of their educational background, physical appearance or other factors) may request higher remuneration (Almeling, 2009).

One egg donor had donated more times than the permitted limit of six donations per donor set by the ICMR. The clinic does not allow donors to exceed this limit (which includes previous donations at different clinics), but it is possible for donors to deliberately withhold information on previous donations in order to continue donating eggs. Clinics have to rely on women to be truthful about the number of donations they have undertaken, as it is impossible to track donations elsewhere. Clinics should consider highlighting to donors the risks involved in donating more than six times, which may encourage them to stay within the permitted limit.

The women in the present study generally had a good understanding of what egg donation is and what the process involves. Most had obtained information from their agent or from clinic staff. That some egg donors appeared to have little information about what their eggs would be used for is a concern. It is possible that these egg donors were aware of this but did not want to mention it during the interview. However, the fact that one donor had questioned why she would need to know what the eggs were for suggests that it is more likely that they did not seek out this information. A minority of egg donors sought their own information using the internet, which was viewed as a convenient and discrete way to gain information, although this option was only available to women of a higher social class who had access to the internet.

Only six women spoke about a possible physical resemblance between themselves and any resultant child; most were unaware that the child could look like them. Surrogates in India have been found to view bodily fluids, such as blood and breast milk, as more important than eggs in shaping a child’s identity, with the claim to motherhood being based upon effort or labour rather than on genetic contributions (Pande, 2009). The connection (or lack thereof) felt between an egg donor and a resultant child is likely to differ in different cultures and contexts (Edwards, 2014). It is important to note that the more educated women in this sample were aware of the link between eggs and resemblance, suggesting that the lack of understanding of most egg donors may relate to their educational background.

The current study recruited egg donors from a single clinic. This ensured that all women who had donated at the clinic were contacted in order to obtain a representative sample and to enable a response rate to be calculated. It also meant that the egg donors could remain anonymous to the interviewer as their full name and contact details were not collected. Being contacted by the clinic may have resulted in some egg donors feeling under pressure to take part. However, some egg donors declined to participate, suggesting that this is not the case. In addition, it was made clear to all egg donors that they did not have to take part and could withdraw from the study if they wished. It is also possible that being invited by the clinic may have influenced the egg donors’ responses, although the fact that some egg donors spoke to the interviewer about issues that the clinic was unaware of, for example, the number of previous donations, suggests that they were speaking openly and truthfully.

A particular strength of this study included the use of in-depth interviews. Furthermore, interviews were conducted by the researcher privately with no member of the clinic present and it was made clear that individual responses would not be discussed with clinic staff, which enabled the egg donors to speak openly about their experiences. Despite this, we cannot rule out the possibility of egg donors responding in a socially desirable way. The interviews were conducted in Hindi and translated for analysis, which may have run the risk of some of the meaning being lost. However, to minimize this risk two researchers were involved in the data analysis. As only egg donors from a single clinic took part in this study, the question of how representative they are of egg donors at other clinics could be posed. A number of egg donors reported that Corion Fertility Clinic was the best clinic they had attended where they felt looked after by the staff. Future studies should examine the experiences of egg donors across a range of different clinics.

Whilst Indian recipients are unlikely to disclose their use of donated eggs to their children, patients from abroad may be more likely to do so, particularly when the child looks visibility different to them and where they are living in a country where parents are encouraged to disclose gamete donation to the child, such as the UK and Australia. Most donors in the current study did not want information about the resultant child, which may be related to their lack of awareness of what egg donation is. Although donors were uninterested in a resultant child, whether or not a child will be uninterested in their donor remains to be seen.
